# miRNA signature of schwannomas: Possible role(s) of “tumor suppressor” miRNAs in benign tumors

**DOI:** 10.18632/oncotarget.251

**Published:** 2011-03-30

**Authors:** Erdogan Pekcan Erkan, Xandra O. Breakefield, Okay Saydam

**Affiliations:** ^1^ Molecular Oncology Research Unit, Division of Oncology, Department of Pediatrics, Medical University of Vienna, Vienna 1090 Austria; ^2^ Departments of Neurology and Radiology, Massachusetts General Hospital, and Neuroscience Program, Harvard Medical School, Boston, Massachusetts, 02129 USA

**Keywords:** miR-7, Tumor suppressor miRNAs, Benign tumors, Schwannomas, Neurofibromatosis 2 (NF2), Ack1

## Abstract

miRNAs have been recently implicated as drivers in several carcinogenic processes, where they can act either as oncogenes or as tumor suppressors. Schwannomas arise from Schwann cells, the myelinating cells of the peripheral nervous system. These benign tumors typically result from loss of the neurofibromatosis type 2 (NF2) tumor suppressor gene. We have recently carried out high-throughput miRNA expression profiling of human vestibular schwannomas using an array representing 407 known miRNAs in order to explore the role of miRNAs in the tumorigenesis of schwannomas. We found that miR-7 functions as a “tumor suppressor” by targeting proteins in three major oncogenic pathways - EGFR, Pak1, and Ack1. Interestingly, in this study, we also observed that several previously described potential tumor suppressor miRNAs that are down-regulated in malignant tumors were up-regulated in schwannomas. Here we discuss the possibility that “tumor suppressor” miRNAs may play a role in the transition stage(s) of cancer from benign to malignant forms.

## MIRNA BIOGENESIS

It has been almost two decades since the discovery of a new class of non-coding RNA molecules by Ambros and colleagues [1]. The term microRNA (miRNA) was coined to describe these short (~21-23 nucleotides long), single-stranded RNA molecules which were later shown to be a key part of post-transcriptional regulatory mechanisms of gene expression in diverse organisms [2,3]. Up to now, >15000 miRNAs have been identified in >100 species (miRbase, Release 16, Sept 2010; http://www.mirbase.org/). Computational predictions suggest that mammalian miRNAs control the activity of up to one-third of known protein-coding genes [4]. miRNA functions are evident from birth to death, encompassing diverse cellular processes, such as cell proliferation, differentiation, development and cell death [5]. Alterations in miRNA biogenesis and/or levels are associated with several disease states, including cancer, autoimmune disorders and neurodegenerative diseases.

**Figure 1:**
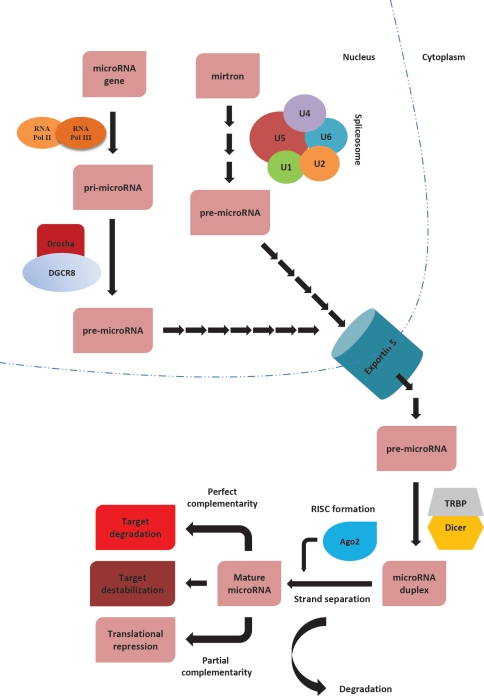
Simplified representative scheme of miRNA biogenesis miRNA biogenesis starts in nucleus, where miRNA genes are transcribed by either RNA polymerase II or III, producing “pri-microRNAs”. Pri-microRNAs are then cleaved by the action of a microprocessor complex, consisting of Drosha-DGCR8, and giving rise to pre-microRNAs. When the length and secondary structure of an intron (mirtron) resembles that of a microRNA, cellular splicing machinery can single-handedly take the place of Drosha processing to produce a pre-microRNA. Exportin-5 mediates the transport of these pre-microRNA molecules into the cytoplasm, where they interact with a number of different proteins to form RNA-induced silencing complex (RISC). Dicer cleaves pre-microRNAs into duplex miRNAs, whereas Argonoute proteins mediate strand selection to produce mature miRNA molecules. The nature of the complementarity between the miRNA and its target determine the ultimate fate of the transcript. If the complementarity is perfect, the target will be cleaved. On the other hand, in case of imperfect complementarity, the result will be repression of translation. Alternatively, miRNAs can also destabilize target RNAs through deadenylation

Owing to the extensive research on miRNA biogenesis in the past decade, we now understand the precise details of how miRNAs are produced within the cell. miRNA biogenesis is spatially organized in two compartments: nuclear and cytoplasmic (Fig. 1). Within the nucleus, RNA polymerase II or III dictates the transcription of miRNA-coding genes to produce “pri-microRNAs”. These pri-microRNA molecules are then cleaved by the action of a microprocessor complex consisting of Drosha, a RNase III class enzyme, and a double stranded RNA-binding protein, DGCR8 (DiGeorge critical region 8), generating so-called pre-microRNAs (~70 nucleotide) [6,7]. Alternatively, transcription of very short intronic sequences (referred to as mirtrons) by RNA polymerase II and further splicing & debranching processes can also produce pre-microRNAs, thereby by-passing the initial cleavage by the microprocessor complex [8]. In either case, resulting pre-microRNA molecules are transferred to cytoplasm through Exportin 5 — Ran-GTP. Within the cytoplasm, another RNase III class enzyme, Dicer, interacts with other double stranded RNA-binding proteins, including Argonaute 2 (Ago2), to form RISC (RNA-induced silencing complex), which binds to pre-microRNA molecules to cleave them into miRNA-duplexes. Following strand selection and separation by Ago2, activated RISC binds to its mRNA target(s) to exert its effect on translation. The ultimate result of miRNA-mediated regulation of gene expression depends on the extent of complementarity between the miRNA and its target sequence (Fig. 1). In case of perfect complementarity, miRNAs will mediate target cleavage. If the complementarity is imperfect, the result will be translational repression [9].

## ONCOMIR AND TUMOR SUPPRESSOR MIRNAS IN CANCER

Considering their roles in cell proliferation and death, it has been suggested that miRNAs could contribute to oncogenesis. Indeed, miRNA profiling studies revealed that dysregulation in miRNA levels are associated with various cancers [reviewed in 11,^12^]. The term “oncomir” has been coined to describe these cancer-associated miRNAs, although some use this term only to describe miRNAs showing oncogenic characteristics. Recent studies have also shown that defects in miRNA processing are also related to cellular transformation and tumor formation [10]. Further support for miRNA-cancer association comes from genome-wide analysis of cancer-associated microRNAs. It has been estimated that almost ~50% of miRNA-coding genes are present in fragile sites and/or cancer-associated regions within the genome [13,14].

Characterization of the chromosome 13q14 deletion in human chronic lymphocytic leukemia (CLL) represented the first indication of involvement of a miRNA in cancer, showing that miR-15 and miR-16, encoded in this chromosomal region, were either lost or downregulated in a majority of CLL patients [15]. Further research on miRNA-cancer association predominantly focused on elucidating differential miRNA expression profiles in different cancers. As a result, we now know that hundreds of miRNA levels are dysregulated in malignant tumors of lung, breast, colon, liver and brain.

Depending on the disease state, expression of specific microRNAs can either be increased or decreased. miRNAs with upregulated expression levels in cancer, often known as “oncomirs”, aid tumor formation. Examples of such oncomirs include: miR-17-92, associated with lung cancer and lymphoma [16,17]; and miR-372 and miR 373, associated with testicular germ cell tumors [18]. On the other hand, miRNAs with downregulated expression levels are regarded as “tumor suppressors”. These molecules have negative effect on tumor formation and development. The let-7 family represents one of the best examples of a tumor suppressor miRNA [19].

There is still a long way to go before deciphering miRNA involvement in cancers will be fully elucidated. Hundreds of candidate miRNAs remain to be characterized as oncomirs or tumor suppressors for different types of tumors. The precise details of miRNAs function in cancer pathogenesis, as well as the diagnostic and therapeutic potential of miRNAs are an active area of research.

## MIRNA REGULATION OF SCHWANNOMAS

In a recent study, we defined the miRNA signature of schwannomas by miRNA microarray expression profiling of human vestibular schwannomas as compared to control nerve sheaths [20]. This signature includes 12 miRNAs that are deregulated in most schwannoma tumor samples. Out of these 12 miRNAs, 8 were confirmed to be significantly upregulated in schwannomas (5-20-fold); and 4 miRNAs to be downregulated (5-12-fold). Based on the relative fold increase, as assessed by qRT-PCR assays, the most upregulated miRNAs in schwannomas were let-7d (about 22-fold), miR-451 (about 17-fold), and miR-23b (about 15-fold). The let-7 family has been the most studied of the potential “tumor suppressor” miRNAs and contains 11 family members (http://microrna.sanger.ac.uk/cgi-bin/sequences/query.pl?terms=let-7; reviewed in 21). This family acts as tumor suppressors to control several oncogenic pathways, including the Ras pathway [22], as well as oncogenes, HMGA2 [23] and c-Myc [24]. Two recent studies support the possible tumor suppressor function of this miRNA family in mouse models of breast and lung cancer, with elevated levels of let-7 inhibiting growth of these tumors [25,26]. In our study, we found that one of the members of the let-7 family, let-7d, was upregulated in schwannomas. Interestingly, we have recently shown in another NF2-related benign tumor, meningiomas, that let-7d, let-7b, and let-7g are also upregulated as compared to the arachnoidal tissue of origin [27]. Interestingly, the second most upregulated miRNA in schwanommas, miR-451 has also recently been shown to function as a potential tumor suppressor miRNA in human gastric and colon cancer cells, with its overexpression decreasing proliferation and increasing response to ionizing radiation in culture [28].

However, in some cases, there appears to be reverse regulation of miRNA levels in malignant versus benign tumors. In human malignant prostate cancers, miR-23a and miR-23b were shown to be downregulated compared to normal prostate tissues [29]. In contrast, in our study miR-23b was found to be upregulated in benign schwannomas. Interestingly, a recent study showed that miR-23b downregulation resulted in increased expression of the protein encoded in one of its target mRNAs, mitochondrial glutaminase [30]. This, in turn, appears to be responsible for increased glutamine catabolism in prostate cancers. This study indicates a novel link between miRNAs, oncogenes and glutamine metabolism, presumably to provide alternative and quick ATP sources of glucose metabolism in cancers [30]. Thus, in schwannomas, elevated miR-23b might also block alternate ATP sources, but in this case serving to reduce the rate of proliferation of these benign tumor cells. Another interesting potential tumor suppressor miRNA in cancer, miR-29 was also found to be upregulated in schwannomas. The downregulation of miR-29 and upregulation of its oncogenic targets, Tcl1 (T-cell leukemia/lymphoma 1), Mcl1 (an anti-apoptotic Bcl-2 family member) and DNA methyltransferase (DNMT3), have been implicated in chronic lymphocytic leukemia, cholangio-carcinoma and lung cancer as a means of blocking tumor cell apoptosis and silencing tumor suppressor genes [31,32]. Again upregulation of this miR29 may serve to attenuate the growth of benign schwannomas. In summary, several previously known downregulated “tumor suppressor miRNAs” in malignant tumors, such as let-7d, miR-451, miR-23a, and miR-29 were found to be upregulated in schwannomas. Based on these observations and given the fact that let-7d, let-7b, and let-7g tumor suppressor miRNAs are also upregulated in benign meningiomas [27], it seems likely that these miRNAs may function at transition stages in cancer, at least for schwannoma and meningioma tumors, between benign and malignant states by differential regulation of certain oncogenic pathways. So far, there has been no any mouse knockout studies for those miRNAs. It will be interesting to see whether loss of any of these malignant tumor suppressor miRNA can initiate tumor formation or promote malignant tumors in mice. Our miRNA profile comparison studies between benign (WHO grade I), atypical (WHO grade II) and malignant (WHO grade III) in meningiomas showed that several tumor suppressor miRNAs such as let-7 family members become downregulated in the transition between a benign state and a malignant state, while potential oncogenic miRNAs such as miR-21 are upregulated in this transition (O. Saydam, unpublished data). It remains to be explored whether these miRNAs are also upregulated in other types of benign tumors during this transition. The status of potential and confirmed targets of these upregulated miRNAs in schwannomas and meningioma, such as members of the Ras family, p53 or other oncogenic pathways also remains to be investigated.

## MIR-7 TAKE ON THREE ONCOGENIC SIGNALING PATHWAYS

In our study [20], we found that miR-7 was one of the most downregulated miRNAs (~9-fold) in schwannomas compared to control nerves. To investigate the possible contribution of miR-7 to schwannoma growth, we performed gain-of-function studies and found that upregulation of miR-7 inhibited schwannoma cell growth both in culture and in a xenograft tumor model *in vivo*. Moreover, overexpression of miR-7 directly targeted and inhibited expression of Ack1, Pak1, and EGFR in schwannoma cells. A significant inverse correlation was also found between miR-7 downregulation and Ack1 and Pak1 upregulation in schwannoma tumor samples compared to control nerve tissue.

Previously known targets for miR-7 include messages for signaling proteins, Pak1 [33] and epidermal growth factor receptor (EGFR) [34], known to be activated in many forms of cancer. Epidermal growth factor receptor is overexpressed, amplified or mutationally activated in glioma, breast, lung, esophageal, and head and neck cancers [35]. Paks play an essential role in a variety of cellular functions including cell division, survival, angiogenesis, growth factor signaling and cell migration [36]. Overexpression of Paks has been detected in many cancers, such as glioma and breast cancer and linked to increased invasion and metastasis [37,38].

A new target for miR-7, found in our study, associated cdc42 kinase 1 (Ack1) is a non-receptor protein tyrosine kinase [39], and the gene encoding Ack1 has been recently shown to be amplified in breast, esophageal, lung, ovarian, pancreatic, and prostate cancer [40]. In a recent study, the role of Ack1 in migration and invasion of breast cancer cells was found to correlate with preservation of EGFR expression *in vitro* [41]. In prostate cancer, Ack1 stimulates prostate tumorigenesis in part by negatively regulating the proapoptotic tumor suppressor, the WW domain containing oxidoreductase (Wwox) [42]. Ack1 interacts with Wwox and triggers its ubiquitination and degradation. The same study also provided evidence supporting an oncogenic role of Ack1 *in vivo* - Ack1 overexpression promoted anchorage-independent growth in culture and tumor growth *in vivo* [42]. It remains to be investigated how upregulation of Ack1 by decreased miR-7 contributes to schwannoma tumorigenesis.

A recent study showed that miR-7 inhibited EGFR and Akt signaling by directly targeting the EGFR mRNA and the 3'UTR of IRS-1 and IRS-2, which function as upstream regulators of the Akt pathway [34,43]. Another study demonstrated that Pak1 mRNA is also a target for miR-7, with upregulation of this miRNA leading to degradation of Pak1 mRNA in transformed HeLa, ZR-75, and HEK 293 cells [33]. Taken together, our results and other reports [33,34] support the function of miR-7 as a potential tumor suppressor in tumors, including malignant gliomas and benign schwannomas. Overexpression and activation of Pak1 has been detected in many cancers [44], including schwannomas [45,46; see review 36]. Since the *NF2* tumor suppressor gene is deleted in most schwannomas and its gene product, merlin inhibits Pak1 activation in several cell lines, including a mouse fibroblastic line, NIH 3T3 and a rat schwannoma cell line, RT4-DP6 [45,46], it seems very probable that Pak1 activation is critical in schwannoma formation. Additional mechanisms to activate Pak1, for example, via downregulation of miR-7 may also contribute to tumorigenesis.

Studies supporting a role for Pak1 [45,46], EGFR [47], and Ack1 [20] activation/overexpression in schwannoma growth, suggests alternative strategies and rationale for the development of new therapies for these tumors based on overexpression of miR-7 or inhibition of Ack1, Pak1, and EGFR pathways. Given the fact that schwannomas, as many other cancers, are not always responsive to anti-EGFR treatment [48], our study suggests that Pak1 and/or Ack1 may prove critical therapeutic targets for schwannomas.
